# Differences in meningococcal disease incidence by health insurance type and among persons experiencing homelessness—United States, 2016–2019

**DOI:** 10.1371/journal.pone.0293070

**Published:** 2023-10-19

**Authors:** Cheryl J. Isenhour, Samuel J. Crowe, Lucy A. McNamara

**Affiliations:** Division of Bacterial Diseases, National Center for Immunization and Respiratory Diseases, Centers for Disease Control and Prevention, Atlanta, Georgia, United States of America; Chiesi USA, UNITED STATES

## Abstract

Meningococcal disease is a serious but rare disease in the United States. Prior publications suggest incidence differs among privately vs publicly-insured persons, and that incidence is higher among persons experiencing homelessness (PEH) than persons not known to be experiencing homelessness (non-PEH). Using insurance claims data for persons aged <1 to 64 years, we calculated meningococcal disease incidence among a population with employer-sponsored commercial insurance and persons enrolled in state Medicaid programs nationwide. We also examined meningococcal disease incidence by PEH status in Medicaid data. From 2016 through 2019, persons who met our study inclusion criteria contributed a total of 84,460,548 person-years (PYs) to our analysis of commercial insurance data and 253,496,622 PYs to our analysis of Medicaid data. Incidence was higher among persons enrolled in Medicaid (0.12 cases per 100,000 PYs) than persons with commercial insurance (0.06 cases per 100,000 PYs). Incidence was 3.17 cases per 100,000 PYs among PEH in Medicaid, 27 times higher than among non-PEH in Medicaid. Understanding the underlying drivers of the higher meningococcal disease incidence among PEH and persons enrolled in Medicaid may inform prevention strategies for populations experiencing a higher burden of disease.

## Introduction

Meningococcal disease, caused by infection with the bacterium *Neisseria meningitidis*, is a rare but serious illness. The disease may be fatal or cause long-term sequelae among survivors, making it crucial to identify and understand risk factors for infection. In the United States, vaccines are available to protect against the most common serogroups. Meningococcal disease incidence has declined in the United States over time to an estimated 0.11 cases per 100,000 persons in 2019, with the highest incidence observed among infants and adolescents [1]. The Advisory Committee on Immunization Practices recommends vaccination of adolescents, college students, travelers to countries where meningococcal disease is endemic, and persons with certain underlying health conditions: human immunodeficiency virus (HIV), complement component deficiencies or use of complement inhibitors, and anatomic or functional asplenia [2]. Exposure to tobacco smoke has also been associated with increased meningococcal disease risk [3]; however, meningococcal vaccination is not routinely recommended for this population.

A previous analysis using health insurance claims data to describe the burden of vaccine-preventable diseases by insurance type in the United States found that meningococcal disease incidence may be as much as 13 times higher among persons enrolled in a public health insurance plan compared to persons with commercial insurance [4]. However, the prior claims-based analysis included data from over 10 years ago (2006–2010), from a subset of anonymous state Medicaid programs, excluded persons less than 19 years of age, and relied on a non-specific definition of meningococcal disease. Furthermore, a recent analysis of enhanced meningococcal disease surveillance data has demonstrated an increased risk among persons experiencing homelessness (PEH), with incidence observed to be 19.8 times higher than among persons not known to be experiencing homelessness (non-PEH) [5]. Neither of these prior analyses were able to ascertain whether the observed higher risk could be attributed to other factors known to impact meningococcal disease risk, such as concurrent underlying health conditions, crowded living conditions, or exposure to tobacco smoke. Gaining a better understanding of the factors contributing to higher incidence among both PEH and the broader Medicaid-insured population is central to the development of prevention strategies, including future vaccine recommendations.

We aimed to assess meningococcal disease incidence among persons with private health insurance, persons with public health insurance, and persons experiencing homelessness; including differences by patient characteristics in each population. We used two large health insurance claims databases to examine annual meningococcal disease incidence, and incidence across the four-year study period, among persons enrolled in employer-sponsored commercial insurance and persons enrolled in all state Medicaid programs. We also described four-year incidence by patient characteristics, including PEH status, household size, and family income level among persons enrolled in Medicaid, to better understand whether the increased risk among PEH is unique, or if other persons of lower socioeconomic status (SES) also experience higher burden of meningococcal disease. For both insured populations, we also calculated incidence among persons with and without claims associated with current tobacco use or underlying conditions for which vaccination is recommended: human immunodeficiency virus (HIV), complement component deficiencies or use of complement inhibitors, and anatomic or functional asplenia.

## Materials and methods

We used data from the Merative™ MarketScan® Commercial Claims and Encounters (CCAE) database and the Centers for Medicaid and Medicare Services (CMS) Transformed Medicaid Statistical Information System (T-MSIS) Analytic Files for this analysis [6, 7]. MarketScan and CMS data include insurance claims from inpatient, outpatient, and pharmacy settings, as well as enrollment data for persons with employer-sponsored health insurance or persons enrolled in state Medicaid programs. The Merative databases include data from approximately 350 commercial payers, while the T-MSIS files include data for persons enrolled in traditional Medicaid and Children’s Health Insurance plans (CHIP), available in all 50 states, and those enrolled in Medicaid expansion plans in states offering that option as part of the 2010 Patient Protection and Affordable Care Act (ACA) [8–10]. Together, Medicaid and CHIP provide health insurance coverage for over 72 million persons, including low-income families, qualified pregnant women, and persons with disabilities. In states that have elected to adopt ACA Medicaid expansion, adults with income at or below 133% of the federal poverty level may also be eligible for coverage [11].

**Fig 1:**
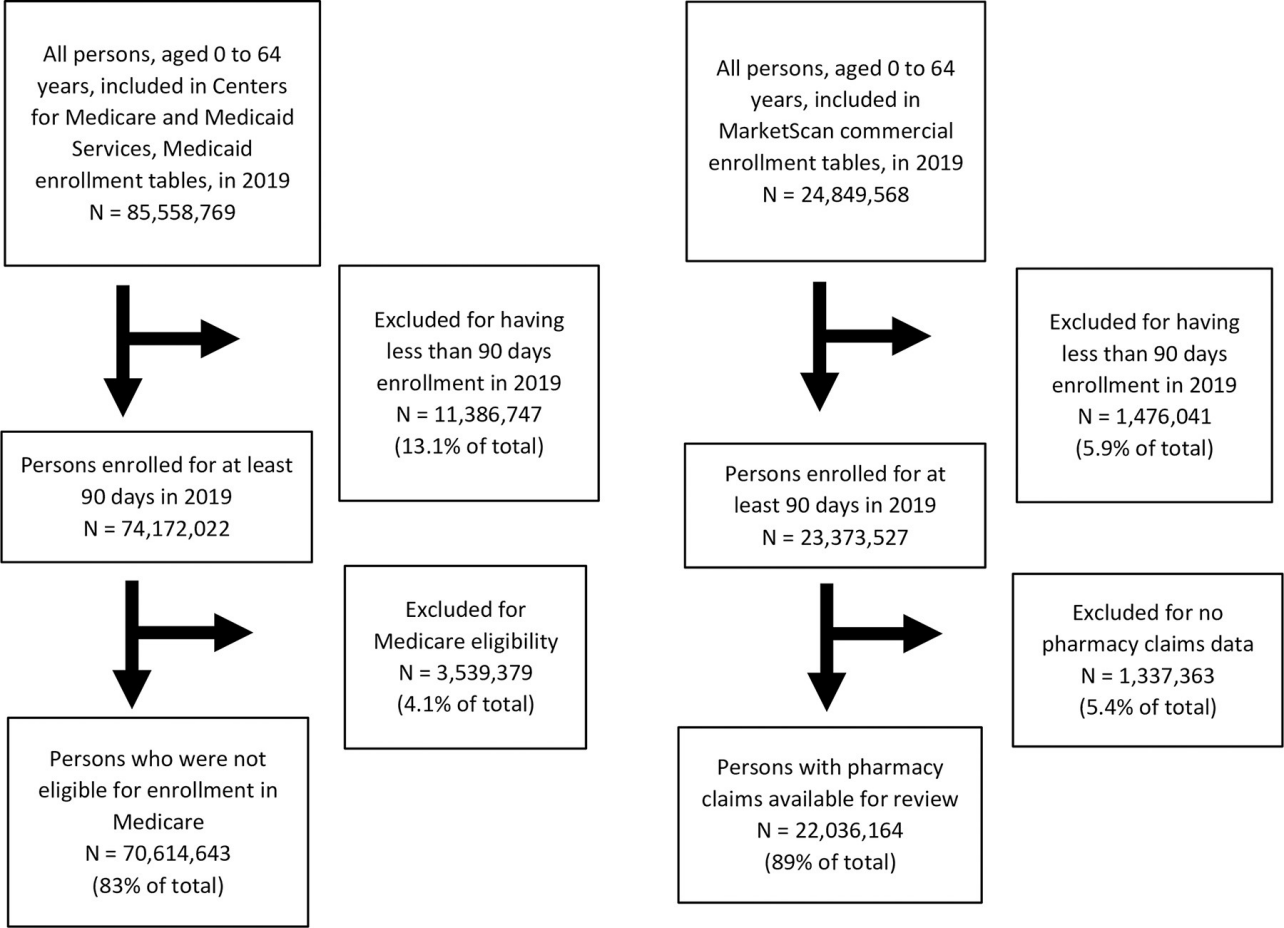
Study selection process for both United States Centers for Medicare and Medicaid Services, Medicaid data, and MarketScan commercial insurance claims data, 2019.

All persons included in the analysis were required to be aged less than 65 years and enrolled in an insurance plan with both medical and prescription drug coverage. We excluded persons from the Medicaid analysis if they were dually eligible for Medicare benefits. Within each calendar year of interest, we retained persons who were enrolled for at least 90 days and did not require enrollment time to be continuous. We allowed individuals to contribute person time to the analysis for each year they met our study criteria. An example of the study population selection process for 2019 is shown for both the MarketScan and Medicaid data (Fig 1).

We identified persons with meningococcal disease as having at least one International Classification of Diseases, Tenth Revision, Clinical Modification (ICD-10-CM) principal diagnosis code on claims from inpatient admissions (S1 Table). We defined PEH status as persons having at least one medical claim with an ICD-10-CM diagnosis code (Z59.0 or Z59.1) indicative of homelessness. We also used diagnosis, procedure, and National Drug Codes to identify persons with underlying conditions for which meningococcal vaccines are recommended, including HIV, sickle cell anemia, complement component deficiency, asplenia, and treatment with either eculizumab or ravulizumab (S2 and S3 Tables). To identify persons with possible exposure to tobacco smoke, we used an extensive list of diagnosis and procedure codes to identify many different types of tobacco use, including codes for documenting the use of cigarettes (S2 and S3 Tables). To classify persons by PEH status, the presence of underlying conditions, or tobacco use, we reviewed claims within each calendar year separately. For persons who met our study criteria in more than one calendar year, this approach allowed us to identify any changes in status from year to year and to assign person time for each group accordingly.

For 2016 through 2019, we calculated annual meningococcal disease incidence per 100,000 person-years (PYs) enrolled, and incidence for the four-year study period, among all persons who met our study criteria in MarketScan and Medicaid data, as well as for both PEH and non-PEH in Medicaid data. For both MarketScan and Medicaid data, we described total time contributed, total persons with meningococcal disease, and meningococcal disease incidence by age group, sex, U.S. census division, insurance plan type (managed care or fee-for-service), tobacco use, and underlying conditions status. We also reported the same metrics by race and ethnicity, family income level, and household size for persons enrolled in Medicaid; these characteristics were not available in MarketScan. For each of the patient characteristics, we used bivariate Poisson regression models to identify significant differences in meningococcal disease incidence versus a reference group, within each population. We reported p-values only for variables that were determined to be significant in each bivariate model at p<0.05 (S4 Table).

Our CMS data use agreement required suppression of all cell sizes smaller than 11. Consequently, certain demographic categories were collapsed, including the creation of ten-year age groups over age 45, combining all underlying conditions of interest, combining family income levels over 100% of the federal poverty level, and combining household sizes over 5 people. We combined Asian, American Indian and Alaska Native, Hawaiian and Pacific Islander, and Multiracial groups to create an other, non-Hispanic race category. Additionally, the cell size limitation prevented us from presenting incidence by characteristics for PEH in Medicaid.

We repeated all incidence calculations after excluding persons with underlying conditions associated with an increased risk of meningococcal disease to better understand the impact of these conditions on the observed differences in incidence among our populations of interest. Finally, we compared four-year incidence reported in MarketScan and Medicaid with national surveillance data collected through the National Notifiable Diseases Surveillance System for the same time period [12]. This analysis of deidentified insurance claims data did not require human subjects review. Analyses were conducted in 2022 using SAS 9.4, Cary, NC.

## Results

From 2016 through 2019, persons who met our study inclusion criteria contributed a total of 84,460,548 person-years to our MarketScan analysis and 253,496,622 person-years to our analysis of Medicaid data (Table 1). The selected Medicaid population skewed younger with persons aged <1 through 19 years contributing over half (58%) of the total person-years in the Medicaid analysis, compared to 35% in MarketScan for the same ages. By U.S. census division, less person time was contributed by persons residing in the Pacific region in MarketScan (10%) relative to Medicaid (21%). In contrast, the South Atlantic region was overrepresented in MarketScan (21%) compared to Medicaid (16.1%). Insurance plan type varied considerably between the two data sources, with most time contributed by persons with a managed care plan in Medicaid (85%) and the inverse in MarketScan (12%). Among persons included in the Medicaid analysis with non-missing data for race and ethnicity, family income level, or household size, 42% of person time was contributed by white, non-Hispanic persons, 65% was contributed by persons at 0 to 100% of the federal poverty level, and 35% was contributed by persons residing in a single-person household.

**Table 1:** Meningococcal disease incidence, per 100,000K person-years, by select patient characteristics, in Centers for Medicare and Medicaid Services, Medicaid data, and MarketScan commercial insurance claims data, 2016–2019.

	CMS Medicaid				MarketScan Commercial Insurance			
	Population Person-years	Cases	Incidence	P-value*	Population Person-years	Cases	Incidence	P-value*
**Total**	253,496,622	311	0.12		84,460,548	49	0.06	
**Age category**								
0 through 4	37,306,574	123	0.33	ref	4,986,547	6	0.12	ref
5 through 9	40,147,946	32	0.08	<0.001	5,277,089	1	0.02	0.087
10 through 14	38,423,099	22	0.06	<0.001	6,032,455	2	0.03	0.114
15 through 19	31,120,248	26	0.08	<0.001	5,942,977	10	0.17	0.516
20 through 24	16,947,686	11	0.06	<0.001	7,185,268	6	0.08	0.527
25 through 29	17,604,252	21	0.12	<0.001	6,018,968	4	0.07	0.358
30 through 34	15,257,225	15	0.10	<0.001	6,298,836	1	0.02	0.061
35 through 39	12,801,438	13	0.10	<0.001	6,624,951	1	0.02	0.055
40 through 44	9,936,837	12	0.12	0.001	6,639,531	2	0.03	0.090
45 through 54	18,115,880	16	0.07	<0.001	14,824,338	8	0.05	0.138
55 through 64	15,835,436	20	0.11	<0.001	14,629,589	8	0.05	0.144
**Sex**	** **							
Male	117,681,505	179	0.15	ref	40,984,144	19	0.05	
Female	135,794,763	132	0.10	<0.001	43,476,405	30	0.07	
Missing	20,354	-	-	-	-	-	-	
**Census Division** **	** **							
New England	10,876,839	16	0.15	ref	2,931,914	2	0.07	ref
Middle Atlantic	35,618,766	43	0.12	0.500	10,589,225	12	0.11	0.506
East North Central	37,215,321	50	0.13	0.752	13,018,718	5	0.04	0.492
West North Central	12,307,892	13	0.11	0.375	4,454,514	2	0.04	0.676
South Atlantic	40,851,307	39	0.10	0.145	17,872,212	3	0.02	0.125
East South Central	16,108,152	17	0.11	0.340	5,221,208	1	0.02	0.300
West South Central	26,654,507	20	0.08	0.045	10,008,822	10	0.10	0.622
Mountain	19,643,515	21	0.11	0.336	4,885,419	3	0.06	0.908
Pacific	54,220,323	92	0.17	0.598	8,547,722	6	0.07	0.972
Missing	-	-	-		6,930,793	5	0.07	0.947
**Insurance Plan Type**	** **							
Managed Care	215,033,476	242	0.11	ref	9,499,227	4	0.04	
Fee-For-Service	38,463,146	69	0.18	0.024	73,331,254	45	0.06	
Missing	-	-	-		1,622,455	-	-	
**Tobacco use**								
No	244,931,624	266	0.11	ref	82,820,652	41	0.05	ref
Yes	8,564,998	45	0.53	<0.001	1,639,896	8	0.49	<0.001
**Underlying condition** ***			** **	** **				
No	252,609,300	296	0.12	ref	84,298,976	47	0.06	ref
Yes	887,322	15	1.69	<0.001	161,572	2	1.24	<0.001
**Race and Ethnicity** ****	** **							
White, non-Hispanic	86,167,714	123	0.14					
Black, non-Hispanic	44,722,305	50	0.11					
Other, non-Hispanic	15,645,163	21	0.13					
Hispanic, all races	54,841,933	51	0.09					
Missing	52,119,507	66	0.13					
**Family Income relative to Federal Poverty Level**								
0 to 100% of FPL	87,890,073	110	0.13	ref				
101 to 400% of FPL	30,714,620	24	0.08	0.037				
Missing	134,891,929	177	0.13	0.687				
**Household size**								
1 person	36,124,536	77	0.21	ref				
2 people	14,095,092	15	0.11	<0.001				
3 people	16,773,529	18	0.11	0.579				
4 people	16,123,739	14	0.09	0.002				
5 or 6 people	15,659,438	13	0.08	0.002				
7 or 8 people	6,872,731	17	0.25	0.009				
Missing	147,847,556	157	0.11	0.014				

*Bivariate Poisson regression models were used to identify significant differences between incidence rates within strata, for each source separately. P-values are reported only for variables that were determined to be significant in each bivariate model at p<0.05.

**New England = CT, MN, MA, NH, RI, VA; Middle Atlantic = NJ, NY, PA; East North Central = IN, IL, MI, OH, WI; West North Central = IA, KS, MN, MO, NB, ND, SD; South Atlantic = DC, DE, FL, GA, MD, NC, SC, VA, WV; East South Central = AL, KY, MS, TN; West South Central = AR, LA, OK, TX; Mountain: AZ, CO, ID, NM, MT, UT, NV, WY; Pacific = AK, CA, HI, OR, WA

***Underlying condition = met at least one definition for an underlying condition associated with higher risk of meningococcal disease; including human immunodeficiency virus, sickle cell anemia, asplenia, complement component deficiency, or treatment with eculizumab or ravulizumab

****Other Race includes Asian, American Indian, Alaskan Native, Hawaiian and Pacific Islander, and Multiracial, non-Hispanic

**Fig 2:**
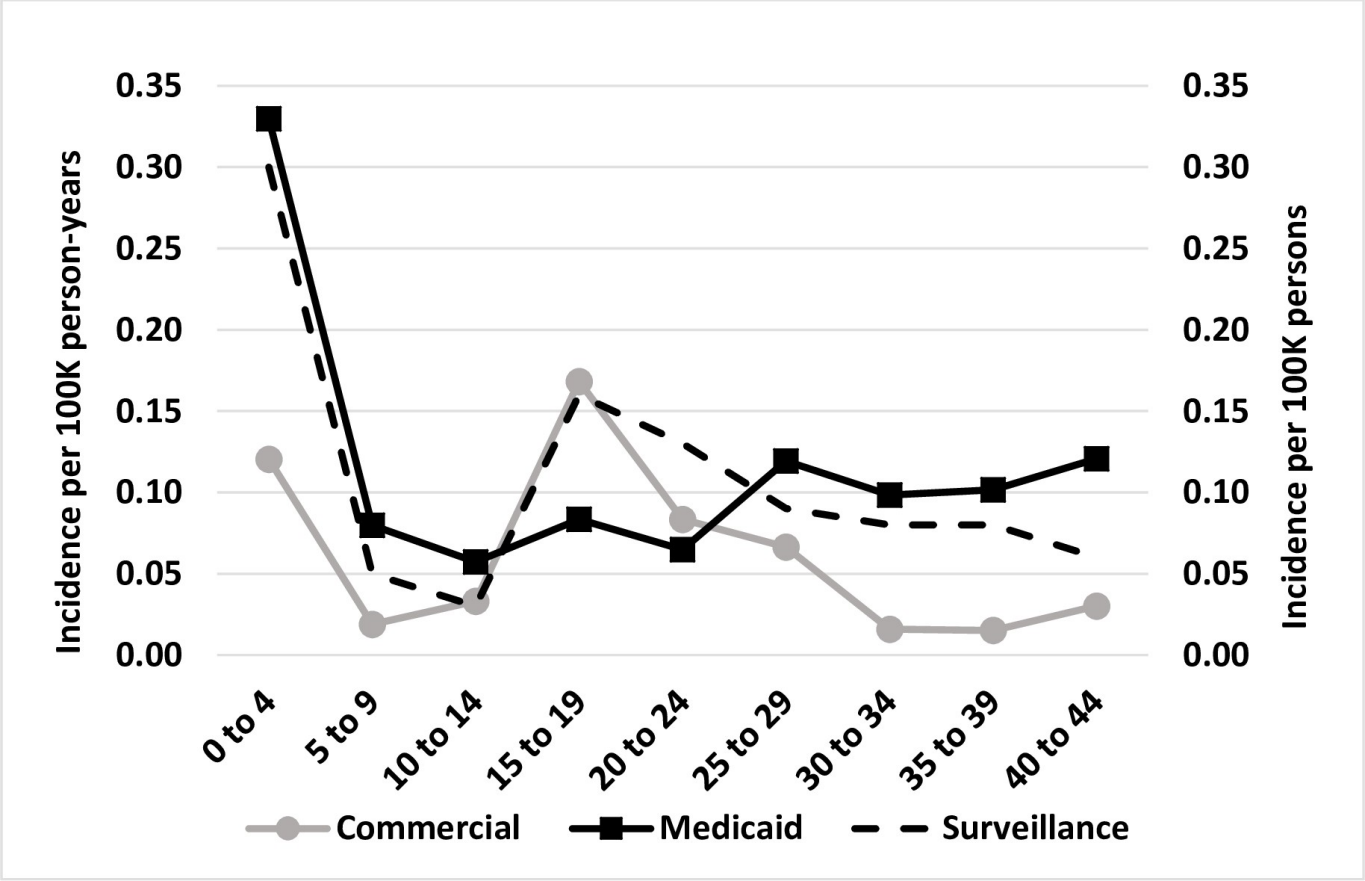
Meningococcal disease incidence per 100,000 person-years among persons in Centers for Medicare and Medicaid Services, Medicaid data and MarketScan commercial insurance claims data, and per 100,000 persons in National Notifiable Diseases Surveillance Systems data, by age group, 2016–2019.

During the four-year study period we identified 49 persons hospitalized with meningococcal disease in MarketScan and 331 in Medicaid. Half of these were aged <1 through 9 years in Medicaid, compared to 14% in MarketScan, while a greater proportion of cases were observed among adolescents aged 15 through 24 years in MarketScan compared to Medicaid (32% and 13% respectively) (Fig 2). Persons with meningococcal disease were predominately male in Medicaid (58%) and female in MarketScan (61%). Among patients in Medicaid with non-missing data for race and ethnicity (254 persons), family income level (134 persons), and household size (154 persons), 50% were non-Hispanic White, 82% had an income of 0 to 100% of the federal poverty level, and 50% were residing in a single-person household.

Meningococcal disease incidence by year varied during the study period from 0.04 to 0.08 cases per 100,000 PYs among persons in MarketScan, and 0.10 to 0.14 among persons in Medicaid (Table 2). Incidence across the four-year study period was twice as high among Medicaid-insured persons as among those in MarketScan, 0.06 and 0.12 cases per 100,000 PYs, respectively (Table 3). After stratifying by age group, incidence among persons enrolled in Medicaid was higher than among those in MarketScan among all age groups except those aged 15 through 24 years. Incidence was 3.17 cases per 100,000 PYs among PEH in Medicaid, approximately 27 times higher than incidence among non-PEH in Medicaid and 53 times higher than the MarketScan population (Table 3).

**Table 2:** Annual meningococcal disease incidence, per 100,000 person-years, by insurance type, in Centers for Medicare and Medicaid Services, Medicaid data, and MarketScan commercial insurance claims data, 2016–2019.

	CMS* Medicaid				MarketScan Commercial			
Year	Total Persons	Person Years	Cases	Incidence	Total Persons	Person Years	Cases	Incidence
2016	71,959,057	63,589,704	91	0.14	24,632,622	22,292,573	18	0.08
2017	72,450,090	64,163,763	62	0.10	23,310,916	21,113,652	9	0.04
2018	71,845,261	63,371,718	81	0.13	23,900,981	21,393,558	14	0.07
2019	70,614,643	62,371,438	77	0.12	22,036,164	19,660,766	8	0.04

*CMS = Centers for Medicare and Medicaid Services

**Table 3:** Meningococcal disease incidence, per 100,000 person-years, by insurance type and homelessness status, in Centers for Medicare and Medicaid Services, Medicaid data, and MarketScan commercial insurance claims data, 2016–2019.

**Population**	**Total Persons**	**Person years**	**Cases**	**Incidence**
MarketScan	93,880,683	84,460,548	49	0.06
CMS* Medicaid	286,869,051	253,496,622	311	0.12
CMS Medicaid Non-PEH**	286,274,266	252,960,594	294	0.12
CMS Medicaid PEH	595,163	536,028	17	3.17

*CMS = Centers for Medicare and Medicaid Services

*PEH = Persons experiencing homelessness

Persons living with HIV, anatomic or functional asplenia, or complement component deficiency, or persons taking complement component inhibitors, had a four-year incidence that was 14 times higher in Medicaid (1.69 cases per 100,000 PYs), and 22 times higher in MarketScan (1.24 cases per 100,000 PYs) relative to those who did not meet our definitions for these underlying condition (0.12 and 0.06 cases per 100,000 PYs, respectively). After excluding persons with these underlying conditions from the analysis, we observed no change in four-year incidence for the base Medicaid and MarketScan populations, but incidence was slightly lower, at 2.71 cases per 100,000 PYs, among PEH without underlying conditions. Even with this slight reduction, incidence among PEH was still 24 times higher relative to incidence among non-PEH in Medicaid, and 45 times higher than in the MarketScan population. We also identified 45 persons with meningococcal disease in Medicaid and 8 persons with meningococcal disease in MarketScan who met our definition of tobacco use during the same year they were hospitalized for meningococcal disease. Incidence was 10 times higher among tobacco users (0.49 cases per 100,000 PYs) than non-tobacco users (0.05 cases per 100,000 PYs) in MarketScan and 5 times higher among tobacco users in Medicaid (0.53 compared to 0.11 cases per 100,000 PYs). More than half of all PEH in Medicaid met our definition of tobacco use (56%), but meningococcal disease incidence was higher among non-tobacco users within this population.

Meningococcal disease incidence varied by patient characteristics (Table 1). Sex and insurance plan type in MarketScan, and race and ethnicity in Medicaid, were determined not to be significant in bivariate Poisson regression models, therefore, results comparing incidence within strata are not reported for these variables. By age group, incidence tracked well with national surveillance data (Fig 2); it was significantly higher among persons aged <1 through 4 years in Medicaid. Incidence was highest among adolescents aged 15 through 19 years in MarketScan, but this difference was not significant. Incidence was also significantly higher among males in Medicaid. Though not statistically significant, By U.S. census division, incidence was highest among persons residing in the Pacific division in Medicaid, and the Middle Atlantic and West South Central divisions in MarketScan. Incidence was significantly higher among persons with family income at 0 to 100% of the federal poverty level, and persons residing in single-person households or households with 7 to 8 people.

## Discussion

Through the analysis of two large insurance claims databases, we found meningococcal disease incidence from 2016 through 2019 to be twice as high among persons enrolled in Medicaid as among persons enrolled in employer-sponsored commercial insurance plans. Our findings revealed a higher burden of disease among all age groups in Medicaid, except among adolescents, for whom incidence was higher in MarketScan. Additional data is needed to fully explain the higher incidence among commercially insured adolescents, but exposures at colleges and universities may play a role. Incidence also varied by patient characteristics, including race and ethnicity, family income level, household size, and PEH status in Medicaid. Incidence among PEH in Medicaid was 27 times higher than among non-PEH in Medicaid, and 53 times higher than among persons in the commercially insured population. These findings add to the growing body of evidence that persons of lower SES, and particularly PEH, are at increased risk for meningococcal disease.

While prior publications have documented a higher burden of both chronic and infectious diseases among persons enrolled in Medicaid relative to national averages [13, 14], we are aware of just one example, by Krishnarajah et al., which specifically describes differences in meningococcal disease incidence by health insurance type [4]. In 2010, they found incidence to be 13 times higher among persons enrolled in 12 anonymous Medicaid programs than among commercially insured persons, at 26.2 and 2.0 cases per 100,000 persons, respectively. These estimates are considerably higher than what we observed in the present analysis, as well as the estimated 0.27 cases per 100,000 persons observed in national surveillance data from 2010 [1]. The differences between our results may be explained, in part, by differences in case identification methods, data sources used, and years of data analyzed. As invasive meningococcal disease is extremely serious, nearly always resulting in hospitalization, we elected to only include patients with principal diagnoses of meningococcal disease on inpatient claims. Therefore, we applied a narrower definition for persons with meningococcal disease than Krishnarajah et al., as they also included claims from other care settings. Our use of data from all state Medicaid programs to calculate incidence, and including all persons aged <1 through 64 years, provided a more complete picture of disease burden in this population, compared with the use of data from adults enrolled in 12 anonymous state Medicaid programs. Moreover, as meningococcal disease incidence has been declining annually in the United States, we would also expect incidence to be somewhat lower in the years we studied than in 2006–2010. These differences aside, both analyses underscore the importance of continuing to examine the variability in meningococcal disease incidence by health insurance type. Additional analyses to assess differences in outcomes following hospitalization for meningococcal disease may also reveal unique challenges to be addressed for both publicly and privately-insured persons.

Although data describing race and ethnicity, as well as the social determinants of health, are usually limited in claims databases, we were able to examine incidence by race and ethnicity, family income level relative to the federal poverty level, and household size for persons enrolled in Medicaid. We found that race and ethnicity were not significantly associated with meningococcal disease incidence. This is in contrast to a publication on national surveillance data from 1995 through 2015, which described meningococcal disease to be 1.4 times higher among Black persons than White persons in the United States. Of note, the authors also observed that the differences in incidence by race have decreased over time, but they did not report on incidence by health insurance type [15]. Incidence was 1.6 times higher among persons at the lowest income level than persons with a higher family income, and by household size we found incidence to be highest among persons living in either a household with 7 or 8 people, or a single-person household. Our findings highlight the need to further examine the impact of these and other factors on meningococcal disease incidence to understand the extent to which persons of low SES are at increased risk. This could include methods for linking health insurance claims with other sources that capture a variety of information on the social determinants of health.

As vaccination is recommended for persons living with HIV, persons with complement component deficiencies or using complement inhibitors, and persons with anatomic or functional asplenia, we sought to examine the impact of these health conditions on meningococcal disease incidence for both insured populations and PEH in Medicaid. As expected, we observed significantly higher meningococcal disease incidence among persons who met our definition of having at least one of these conditions. However, removing these individuals from our analyses did not impact the four-year incidence for the base populations in either MarketScan or Medicaid. Incidence among PEH in Medicaid decreased slightly but was still 24 times higher relative to non-PEH and 45 times higher than the MarketScan population, suggesting that the increased burden of meningococcal disease among PEH cannot be solely explained by a higher prevalence of these underlying conditions.

Our finding that meningococcal disease incidence among PEH enrolled in Medicaid was 27 times higher than among non-PEH is consistent with a recent analysis of enhanced meningococcal disease surveillance data from the same period, which found that incidence was 19.8 times higher among PEH than non-PEH nationally, or 24.6 times higher among persons aged 18 or more years [5]. Of note, this article also described outbreaks among PEH, including 10 meningococcal disease outbreak cases identified among PEH in Boston, Massachusetts, from 2016 through 2019. PEH included in this outbreak may have qualified for enrollment in Massachusetts Medicaid programs during the study period and may have contributed to the relatively high incidence of meningococcal disease observed among PEH in the present analysis.

Steps to fully understand and address the factors underlying increased meningococcal disease risk among PEH are necessary to prevent this vulnerable population from continuing to experience higher rates of this devastating disease; in addition, the potential impact of meningococcal vaccination for this population should be considered. Since our analysis suggests that the presence of certain underlying health conditions may contribute to, but does not fully explain, the increased risk of meningococcal disease among PEH, ensuring PEH have reliable access to the appropriate health care for managing underlying conditions is crucial for the overall health of this population and reduction of infectious disease risk. Additionally, exposure to crowded living conditions has been associated with increased risk of meningococcal disease [2], which is an important consideration for homeless shelters as some meningococcal disease outbreaks have included PEH who visited these facilities [5]. While PEH experience higher rates of vaccine-preventable diseases than the general population, it can be challenging to vaccinate PEH for several reasons, including lack of access to preventative health care, mistrust of providers, and lack of information about infectious disease risk or the importance of vaccination [16–18]. Despite these challenges, there is an existing recommendation to vaccinate PEH against Hepatitis A [19]. If meningococcal vaccines were recommended for all PEH, strategies to implement the recommendation would need to be carefully tailored to the unique needs of this population. This could include approaches shown to be successful in other vaccine campaigns among PEH, such as working closely with trusted organizations serving this population and providing convenient access to vaccine in the communities where PEH reside [18, 20].

Exposure to tobacco smoke has also been associated with increased risk of meningococcal disease, both among persons who smoke tobacco products and non-smokers residing in the home with a person who smokes [3]. Among persons who met our definition of tobacco use, meningococcal disease incidence was 10 times higher in MarketScan and 5 times higher among persons enrolled in Medicaid compared with non-tobacco users. Tobacco use, including smoking, has declined over time in the United States to an estimated 19% of adults in 2020, but varies by demographic characteristics, including health insurance type [21]. Current tobacco use prevalence is estimated to be 16.4% among commercially insured persons, and 28.6% among persons enrolled in Medicaid; the rate of decline has also been slow among persons enrolled in Medicaid [21, 22]. This represents a substantial portion of the population who are potentially at increased risk of meningococcal disease, particularly among persons enrolled in Medicaid. There are currently no recommendations for routine meningococcal vaccination of persons exposed to tobacco smoke. In the absence of vaccine recommendations for this population, efforts to promote smoking cessation interventions and to educate tobacco smokers about meningococcal disease could be considered.

Our analysis is subject to certain limitations. First, while national Medicaid data includes all persons enrolled in Medicaid or CHIP plans, MarketScan is not generalizable to the entire U.S. population of persons with employer-sponsored commercial insurance. Second, claims data are collected for billing purposes and may not include all information that would be useful for further understanding risk factors for disease, such as complete race and ethnicity or other SES data and laboratory test results with serogroup information for persons with meningococcal disease. Third, coding errors, or healthcare encounters that did not generate a claim for reimbursement, could result in misclassification of PEH, current tobacco use, or underlying health conditions status. As meningococcal disease typically results in severe illness, and we have identified persons with principal diagnoses from inpatient claims, misclassification by meningococcal disease status, though possible, is less likely. Fourth, the diagnosis codes used for identifying homelessness may not be used as routinely as other codes documented by providers for reimbursement on claims. Although use of these codes has been increasing over time in Medicare, and among Medicaid claims in New York, the extent to which they are routinely used among providers treating patients across all state Medicaid programs is not well described [23, 24]. An analysis of Veterans Affairs (VA) administrative data found that the sole use of ICD codes for homelessness did not identify all PEH in their population, and described different usage patterns for these codes across VA facilities, suggesting that the codes we selected may be specific, but not sensitive for determining PEH status [25]. Fifth, we required persons in the study to be enrolled for a minimum of 90 days in each calendar year of interest. Any healthcare encounters that occurred when a person was not enrolled would not be available for inclusion in our analysis. Sixth, Medicaid T-MSIS data quality, including enrollment or claims reporting, varies by state and may have impacted our study selection process for this population [26]. Finally, our data use agreement prohibiting publication of small cell sizes prevented us from fully describing the characteristics and underlying conditions status of PEH in the Medicaid population.

Despite these limitations, our analysis is the first to describe meningococcal disease incidence using national CMS Medicaid claims data, including PEH enrolled in Medicaid. Assessing disease incidence by various characteristics and comparing findings with nationally reported surveillance data are notable strengths of this analysis.

## Conclusions

Although meningococcal disease in the United States is uncommon, this illness is devastating for patients and their families. While much is known about some occupations, health conditions, and behaviors that are associated with increased risk of infection, disparities by socioeconomic status are emerging. Continuing to utilize novel data sources and approaches to better understand the underlying drivers of these disparities, including social determinants of health, is crucial for informing strategies to mitigate risk among PEH and other persons of lower SES. Additional studies to evaluate the potential cost and public health impact of recommending meningococcal vaccinations for these populations experiencing a higher burden of disease will also be important for guiding future policy decisions.

## Supporting information

S1 Table International Classification of Diseases, 10th Revision, Clinical Modification diagnosis codes used to identify meningococcal disease in medical claims data. (DOCX)

S2 Table Administrative codes used to define select medical conditions or drugs in claims data. (DOCX)

S3 Table Definitions used to identify select medical conditions in claims data. (DOCX)

S4 Table Output from bivariate Poisson regression models. (DOCX)
