# Supplementary material for: Differences in meningococcal disease incidence by health insurance type and among persons experiencing homelessness—United States, 2016–2019
Source: PLoS One. 2023 Oct 19;18(10):e0293070. doi: 10.1371/journal.pone.0293070 (PMC10586599; doi:10.1371/journal.pone.0293070)
Supplement: S1 Table — (DOCX) [file pone.0293070.s001.docx]

| **S1 Table**: International Classification of Diseases, 10th Revision, Clinical Modification diagnosis codes used to identify meningococcal disease in medical claims data | |
| --- | --- |
|  |  |
| **Code** | **Description** |
| A39.0 | Meningococcal meningitis |
| A39.1 | Waterhouse-Friderichsen syndrome |
| A39.2 | Acute meningococcemia |
| A39.3 | Chronic meningococcemia |
| A39.4 | Meningococcemia, unspecified |
| A39.50 | Meningococcal carditis, unspecified |
| A39.51 | Meningococcal endocarditis |
| A39.52 | Meningococcal myocarditis |
| A39.53 | Meningococcal pericarditis |
| A39.81 | Meningococcal encephalitis |
| A39.82 | Meningococcal retrobulbar neuritis |
| A39.83 | Meningococcal arthritis |
| A39.84 | Post meningococcal arthritis |
| A39.89 | Other meningococcal infections |
| A39.9 | Meningococcal infection, unspecified |
|  |  |
|  |  |
