# Supplementary material for: Differences in meningococcal disease incidence by health insurance type and among persons experiencing homelessness—United States, 2016–2019
Source: PLoS One. 2023 Oct 19;18(10):e0293070. doi: 10.1371/journal.pone.0293070 (PMC10586599; doi:10.1371/journal.pone.0293070)
Supplement: S2 Table — (DOCX) [file pone.0293070.s002.docx]

| **S2 Table:**  Administrative codes used to define select medical conditions or drugs in claims data | | | |
| --- | --- | --- | --- |
|  |  |  |  |
| **Condition or drug** | **Code Type** | **Code** | **Description** |
| Human Immunodeficiency Virus or Acquired Immunodeficiency Syndrome | International Classification of Diseases, 10th Revision, Clinical Modification Codes (ICD10) | B20 | Human immunodeficiency virus [HIV] disease acquired immunodeficiency syndrome |
|  |  | B97.35 | Human immunodeficiency virus, type 2 [HIV 2] as the cause of diseases classified elsewhere |
|  |  | O98.711 | Human immunodeficiency virus [HIV] disease complicating pregnancy, first trimester |
|  |  | O98.712 | Human immunodeficiency virus [HIV] disease complicating pregnancy, second trimester |
|  |  | O98.713 | Human immunodeficiency virus [HIV] disease complicating pregnancy, third trimester |
|  |  | O98.719 | Human immunodeficiency virus [HIV] disease complicating pregnancy, unspecified trimester |
|  |  | O98.72 | Human immunodeficiency virus [HIV] disease complicating childbirth |
|  |  | O98.73 | Human immunodeficiency virus [HIV] disease complicating the puerperium |
|  |  | Z21 | Asymptomatic human immunodeficiency virus [HIV] infection status |
| Sickle Cell Disease | ICD10 | D57.00 | Hb-SS disease with crisis, unspecified |
|  |  | D57.01 | Hb-SS disease with acute chest syndrome |
|  |  | D57.02 | Hb-SS disease with splenic sequestration |
|  |  | D57.03 | Hb-SS disease with cerebral vascular involvement |
|  |  | D57.09 | Hb-SS disease with crisis with other specified complication |
|  |  | D57.1 | Sickle-cell disease without crisis |
|  |  | D57.1 | Sickle-cell disease without crisis |
|  |  | D57.20 | Sickle-cell/Hb-C disease without crisis |
|  |  | D57.211 | Sickle-cell/Hb-C disease with acute chest syndrome |
|  |  | D57.212 | Sickle-cell/Hb-C disease with splenic sequestration |
|  |  | D57.213 | Sickle-cell/Hb-C disease with cerebral vascular involvement |
|  |  | D57.218 | Sickle-cell/Hb-C disease with crisis with other specified complication |
|  |  | D57.219 | Sickle-cell/Hb-C disease with crisis, unspecified |
|  |  | D57.40 | Sickle-cell thalassemia without crisis |
|  |  | D57.411 | Sickle-cell thalassemia with acute chest syndrome |
|  |  | D57.412 | Sickle-cell thalassemia with splenic sequestration |
|  |  | D57.413 | Sickle-cell thalassemia, unspecified, with cerebral vascular involvement |
|  |  | D57.418 | Sickle-cell thalassemia, unspecified, with crisis with other specified complication |
|  |  | D57.419 | Sickle-cell thalassemia with crisis, unspecified |
|  |  | D57.42 | Sickle-cell thalassemia beta zero without crisis |
|  |  | D57.431 | Sickle-cell thalassemia beta zero with acute chest syndrome |
|  |  | D57.432 | Sickle-cell thalassemia beta zero with splenic sequestration |
|  |  | D57.433 | Sickle-cell thalassemia beta zero with cerebral vascular involvement |
|  |  | D57.438 | Sickle-cell thalassemia beta zero with crisis with other specified complication |
|  |  | D57.439 | Sickle-cell thalassemia beta zero with crisis, unspecified |
|  |  | D57.44 | Sickle-cell thalassemia beta plus without crisis |
|  |  | D57.451 | Sickle-cell thalassemia beta plus with acute chest syndrome |
|  |  | D57.452 | Sickle-cell thalassemia beta plus with splenic sequestration |
|  |  | D57.453 | Sickle-cell thalassemia beta plus with cerebral vascular involvement |
|  |  | D57.458 | Sickle-cell thalassemia beta plus with crisis with other specified complication |
|  |  | D57.459 | Sickle-cell thalassemia beta plus with crisis, unspecified |
|  |  | D57.80 | Other sickle-cell disorders without crisis |
|  |  | D57.811 | Other sickle-cell disorders with acute chest syndrome |
|  |  | D57.812 | Other sickle-cell disorders with splenic sequestration |
|  |  | D57.818 | Other sickle-cell disorders with crisis with other specified complication |
|  |  | D57.819 | Other sickle-cell disorders with crisis, unspecified |
| Complement deficiency | ICD10 | D841 | Defects in the complement system |
| Asplenia | ICD10 | 07BP0ZZ | Excision of Spleen, Open Approach |
|  |  | 07BP3ZZ | Excision of Spleen, Percutaneous Approach |
|  |  | 07BP4ZZ | Excision of Spleen, Percutaneous Endoscopic Approach |
|  |  | 07TP0ZZ | Resection of Spleen, Open Approach |
|  |  | 07TP4ZZ | Resection of Spleen, Percutaneous Endoscopic Approach |
|  |  | Q89.01 | Asplenia (congenital) |
|  |  | Z90.81 | Acquired absence of spleen |
|  | Current Procedural Terminology (CPT) | 38100 | Splenectomy; total |
|  |  | 38102 | Splenectomy; total, en bloc for extensive disease |
|  |  | 38120 | Laparoscopy, surgical, splenectomy |
| Eculizumab | Healthcare Common Procedure Coding System (HCPCS) | J1300 | Injection, eculizumab, 10 mg |
|  |  | C9236 | Injection, eculizumab, 10 mg |
|  | National Drug Code (NDC) | 25682-0001-01 | Solution, 300mg/30ml |
| Ravulizumab | HCPCS | J1303 | Injection, ravulizumab-cwvz, 10 mg |
|  | NDC | 25682-0022-01 | Injection, eculizumab -solution, 300mg/30ml |
|  |  | 25682-0025-01 | Injection, eculizumab - solution, 300mg/3ml |
|  |  | 25682-0028-01 | Injection, eculizumab - solution, 1100mg/11ml |
| Tobacco use | ICD11 | F17 | Nicotine dependence |
|  |  | F17.2 | Nicotine dependence |
|  |  | F17.20X | Nicotine dependence, unspecified |
|  |  | F17.200 | Nicotine dependence, unspecified, uncomplicated |
|  |  | F17.201 | Nicotine dependence, unspecified, in remission |
|  |  | F17.203 | Nicotine dependence unspecified, with withdrawal |
|  |  | F17.208 | Nicotine dependence unspecified, with withdrawal, with other nicotine-induced disorders |
|  |  | F17.209 | Nicotine dependence unspecified, with withdrawal, with unspecified nicotine-induced disorders |
|  |  | F17.21 | Nicotine dependence, cigarettes |
|  |  | F17.210 | Nicotine dependence, cigarettes, uncomplicated |
|  |  | F17.211 | Nicotine dependence, cigarettes, in remission |
|  |  | F17.213 | Nicotine dependence, cigarettes, with withdrawal |
|  |  | F17.218 | Nicotine dependence, cigarettes, with other nicotine-induced disorders |
|  |  | F17.219 | Nicotine dependence, cigarettes, with unspecified nicotine-induced disorders |
|  |  | F17.22 | Nicotine dependence, chewing tobacco |
|  |  | F17.220 | Nicotine dependence, chewing tobacco, uncomplicated |
|  |  | F17.221 | Nicotine dependence, chewing tobacco, in remission |
|  |  | F17.223 | Nicotine dependence, chewing tobacco, with withdrawal |
|  |  | F17.228 | Nicotine dependence, chewing tobacco, with other nicotine-induced disorders |
|  |  | F17.229 | Nicotine dependence, chewing tobacco, with unspecified nicotine-induced disorders |
|  |  | F17.29 | Nicotine dependence, other tobacco product |
|  |  | F17.290 | Nicotine dependence, other tobacco product, uncomplicated |
|  |  | F17.291 | Nicotine dependence, other tobacco product, in remission |
|  |  | F17.293 | Nicotine dependence, other tobacco product, with withdrawal |
|  |  | F17.298 | Nicotine dependence, other tobacco product, with other nicotine-induced disorders |
|  |  | F17.299 | Nicotine dependence, other tobacco product, with unspecified nicotine-induced disorders |
|  |  | O99.33 | Tobacco use disorder complicating pregnancy, childbirth, and the puerperium |
|  |  | O99.330 | Smoking (tobacco) complicating pregnancy, unspecified trimester |
|  |  | O99.331 | Smoking (tobacco) complicating pregnancy, first trimester |
|  |  | O99.332 | Smoking (tobacco) complicating pregnancy, second trimester |
|  |  | O99.333 | Smoking (tobacco) complicating pregnancy, third trimester |
|  |  | O99.334 | Smoking (tobacco) complicating childbirth |
|  |  | O99.335 | Smoking (tobacco) complicating the puerperium |
|  |  | T65.21 | Toxic effect of chewing tobacco |
|  |  | T65.211 | Toxic effect of chewing tobacco, accidental (unintentional) |
|  |  | T65.211A | Toxic effect of chewing tobacco, accidental (unintentional), initial encounter |
|  |  | T65.212 | Toxic effect of chewing tobacco, intentional self-harm |
|  |  | T65.212A | Toxic effect of chewing tobacco, intentional self-harm, initial encounter |
|  |  | T65.213 | Toxic effect of chewing tobacco, assault |
|  |  | T65.213A | Toxic effect of chewing tobacco, assault, initial encounter |
|  |  | T65.214 | Toxic effect of chewing tobacco, undetermined |
|  |  | T65.214A | Toxic effect of chewing tobacco, undetermined, initial encounter |
|  |  | T65.22 | Toxic effect of tobacco cigarettes |
|  |  | T65.221 | Toxic effect of tobacco cigarettes, accidental (unintentional) |
|  |  | T65.221A | Toxic effect of tobacco cigarettes, accidental (unintentional), initial encounter |
|  |  | T65.222 | Toxic effect of tobacco cigarettes, intentional self-harm |
|  |  | T65.222A | Toxic effect of tobacco cigarettes, intentional self-harm, initial encounter |
|  |  | T65.223 | Toxic effect of tobacco cigarettes, assault |
|  |  | T65.223A | Toxic effect of tobacco cigarettes, assault, initial encounter |
|  |  | T65.224 | Toxic effect of tobacco cigarettes, undetermined |
|  |  | T65.224A | Toxic effect of tobacco cigarettes, undetermined, initial encounter |
|  |  | T65.29 | Toxic effect of other tobacco and nicotine |
|  |  | T65.291 | Toxic effect of other tobacco and nicotine, accidental (unintentional) |
|  |  | T65.291A | Toxic effect of other tobacco and nicotine, accidental (unintentional), initial encounter |
|  |  | T65.292 | Toxic effect of other tobacco and nicotine, intentional self-harm |
|  |  | T65.292A | Toxic effect of other tobacco and nicotine, intentional self-harm, initial encounter |
|  |  | T65.293 | Toxic effect of other tobacco and nicotine, assault |
|  |  | T65.293A | Toxic effect of other tobacco and nicotine, assault, initial encounter |
|  |  | T65.294 | Toxic effect of other tobacco and nicotine, undetermined |
|  |  | T65.294A | Toxic effect of other tobacco and nicotine, undetermined, initial encounter |
|  |  | V15.82 | Personal history of tobacco use, presenting hazards to health |
|  |  | Z72.0 | Tobacco use |
|  |  | Z87.891 | Personal history of nicotine dependence |
|  | CPT | 99406 | Smoking and tobacco use cessation counseling visit; intermediate, greater than 3 minutes up to 10 minutes |
|  |  | 99407 | Smoking and tobacco use cessation counseling visit; intensive, greater than 10 minutes |
|  | HCPCS | 1034F | Current tobacco smoker |
|  |  | 1035F* | Current smokeless tobacco user |
|  |  | G9276 | Documentation that patient is a current tobacco user |
|  |  | G9458 | Patient documented as tobacco user and received tobacco cessation intervention |
|  |  | G9792 | Most recent tobacco status is not tobacco free |
|  |  | G9902 | Patient screened for tobacco use and identified as a tobacco user |
