# Supplementary material for: Differences in meningococcal disease incidence by health insurance type and among persons experiencing homelessness—United States, 2016–2019
Source: PLoS One. 2023 Oct 19;18(10):e0293070. doi: 10.1371/journal.pone.0293070 (PMC10586599; doi:10.1371/journal.pone.0293070)
Supplement: S3 Table — (DOCX) [file pone.0293070.s003.docx]

| **S3 Table:** Definitions used to identify select medical conditions in claims data | |
| --- | --- |
|  |  |
| Condition or drug | Definition |
| Human Immunodeficiency Virus (HIV) or Acquired Immunodeficiency Syndrome* | At least one inpatient, or two other non-drug claims, with HIV diagnosis codes |
| Sickle Cell Disease* | Three or more non-drug claims with sickle cell diagnosis codes |
| Complement deficiency** | At least one inpatient, or two outpatient claims with complement diagnosis codes separated by 30 or more days |
| Asplenia*** | At least one inpatient, or two outpatient claims with complement diagnosis codes separated by 30 or more days, or at least one procedure code for splenectomy |
| Eculizumab and Ravulizumab | At least one medical or pharmacy claim with procedure or national drug codes for either drug |
| Tobacco use* | At least one inpatient, or two other non-drug claims, with diagnosis codes, or one procedure code claim for tobacco use |
| *Adapted from Centers for Medicare and Medicaid Services Chronic Conditions Categories as described at https://www2.ccwdata.org/web/guest/condition-categories-other | |
| **Adapted from Marshall GS, Ghaswalla PK, Bengtson LGS, Buikema AR, Bancroft T, Koep E, Novy P, Hogea CS. *Low Meningococcal Vaccination Rates Among Patients With Newly Diagnosed Complement Component Deficiencies in the United States*. Clin Infect Dis. 2022 | |
| ***Adapted from Ghaswalla PK, Bengtson LGS, Marshall GS, Buikema AR, Bancroft T, Schladweiler KM, Koep E, Novy P, Hogea CS. *Meningococcal vaccination in patients with newly diagnosed asplenia in the United States*. Vaccine. 2021 | |
