# Supplementary material for: Differences in meningococcal disease incidence by health insurance type and among persons experiencing homelessness—United States, 2016–2019
Source: PLoS One. 2023 Oct 19;18(10):e0293070. doi: 10.1371/journal.pone.0293070 (PMC10586599; doi:10.1371/journal.pone.0293070)
Supplement: S4 Table — (DOCX) [file pone.0293070.s004.docx]

| **S4. Table:** Output from bivariate Poisson regression models | | |  |  |  |  |  |  |  |  |  |  |
| --- | --- | --- | --- | --- | --- | --- | --- | --- | --- | --- | --- | --- |
|  |  |  |  |  |  |  |  |  |  |  |  |  |
| **Likelihood ratio statistics for Type 3 analyses** | | | |  |  |  |  |  |  |  |  |  |
| **Variable** | **Degrees of freedom** | **Chi-Square** | **Pr > ChiSq** |  |  |  |  |  |  |  |  |  |
| **Medicaid** | | | |  |  |  |  |  |  |  |  |  |
| Age group | 10 | 125.6 | <.0001 |  |  |  |  |  |  |  |  |  |
| Sex | 1 | 15.45 | <.0001 |  |  |  |  |  |  |  |  |  |
| Census division | 8 | 19.15 | 0.0141 |  |  |  |  |  |  |  |  |  |
| Insurance plan type | 1 | 4.79 | 0.0286 |  |  |  |  |  |  |  |  |  |
| Tobacco use | 1 | 66.04 | <.0001 |  |  |  |  |  |  |  |  |  |
| Underlying condition | 1 | 51.51 | <.0001 |  |  |  |  |  |  |  |  |  |
| Race and ethnicity | 4 | 7.66 | 0.105 |  |  |  |  |  |  |  |  |  |
| Income level | 2 | 6.54 | 0.0381 |  |  |  |  |  |  |  |  |  |
| Household size | 6 | 34.65 | <.0001 |  |  |  |  |  |  |  |  |  |
| **MarketScan** | | | |  |  |  |  |  |  |  |  |  |
| Age group | 10 | 21.02 | 0.0209 |  |  |  |  |  |  |  |  |  |
| Sex | 1 | 1.88 | 0.1699 |  |  |  |  |  |  |  |  |  |
| Census division | 9 | 17.59 | 0.0403 |  |  |  |  |  |  |  |  |  |
| Insurance plan type | 1 | 0.58 | 0.4467 |  |  |  |  |  |  |  |  |  |
| Tobacco use | 1 | 20.48 | <.0001 |  |  |  |  |  |  |  |  |  |
| Underlying condition | 1 | 8.5 | 0.0035 |  |  |  |  |  |  |  |  |  |
| **Least squares means output from bivariate models** | | | | | | | | | | | | |
| **Medicaid** | | | | | | | | | | | | |
| **Variable** | **Reference group** | **Comparison group** | **Estimate** | **Standard Error** | **z Value** | **Pr > \|z\|** | **Alpha** | **Lower** | **Upper** | **Exponentiated** | **Exponentiated Lower** | **Exponentiated Upper** |
| Age group | 1 | 2 | -1.4199 | 0.1984 | -7.15 | <.0001 | 0.05 | -1.8088 | -1.0309 | 0.2418 | 0.1639 | 0.3567 |
|  |  | 3 | -1.7506 | 0.2315 | -7.56 | <.0001 | 0.05 | -2.2043 | -1.2969 | 0.1737 | 0.1103 | 0.2734 |
|  |  | 4 | -1.3728 | 0.2159 | -6.36 | <.0001 | 0.05 | -1.7958 | -0.9497 | 0.2534 | 0.166 | 0.3869 |
|  |  | 5 | -1.6253 | 0.3147 | -5.16 | <.0001 | 0.05 | -2.2421 | -1.0084 | 0.1969 | 0.1062 | 0.3648 |
|  |  | 6 | -1.0166 | 0.2361 | -4.31 | <.0001 | 0.05 | -1.4794 | -0.5539 | 0.3618 | 0.2278 | 0.5747 |
|  |  | 7 | -1.21 | 0.2735 | -4.42 | <.0001 | 0.05 | -1.746 | -0.674 | 0.2982 | 0.1745 | 0.5097 |
|  |  | 8 | -1.1776 | 0.2916 | -4.04 | <.0001 | 0.05 | -1.7492 | -0.606 | 0.308 | 0.1739 | 0.5455 |
|  |  | 9 | -1.0044 | 0.3024 | -3.32 | 0.0009 | 0.05 | -1.5971 | -0.4116 | 0.3663 | 0.2025 | 0.6626 |
|  |  | 10 | -0.9595 | 0.2411 | -3.98 | <.0001 | 0.05 | -1.4321 | -0.487 | 0.3831 | 0.2388 | 0.6145 |
| Sex | Male | Female | -0.4477 | 0.1147 | -3.9 | <.0001 | 0.05 | -0.6726 | -0.2229 | 0.6391 | 0.5104 | 0.8002 |
| Census division | New England | Middle Atlantic | -0.1976 | 0.2928 | -0.67 | 0.4998 | 0.05 | -0.7716 | 0.3763 | 0.8207 | 0.4623 | 1.4569 |
|  |  | East North Central | -0.09065 | 0.2872 | -0.32 | 0.7523 | 0.05 | -0.6536 | 0.4723 | 0.9133 | 0.5202 | 1.6037 |
|  |  | West North Central | -0.3312 | 0.3734 | -0.89 | 0.375 | 0.05 | -1.0631 | 0.4006 | 0.718 | 0.3454 | 1.4927 |
|  |  | South Atlantic | -0.4323 | 0.2969 | -1.46 | 0.1453 | 0.05 | -1.0142 | 0.1496 | 0.649 | 0.3627 | 1.1613 |
|  |  | East South Central | -0.3321 | 0.3483 | -0.95 | 0.3404 | 0.05 | -1.0148 | 0.3506 | 0.7174 | 0.3625 | 1.4199 |
|  |  | West South Central | -0.6732 | 0.3354 | -2.01 | 0.0447 | 0.05 | -1.3306 | -0.01579 | 0.5101 | 0.2643 | 0.9843 |
|  |  | Mountain | -0.3192 | 0.3318 | -0.96 | 0.3361 | 0.05 | -0.9696 | 0.3312 | 0.7267 | 0.3792 | 1.3927 |
|  |  | Pacific | 0.1428 | 0.2709 | 0.53 | 0.5981 | 0.05 | -0.3881 | 0.6737 | 1.1535 | 0.6783 | 1.9614 |
| Insurance Plan Type | Managed care | Fee-for-service | 0.3265 | 0.1442 | 2.26 | 0.0236 | 0.05 | 0.04384 | 0.6092 | 1.3861 | 1.0448 | 1.8389 |
| Tobacco use | No | Yes | 1.5765 | 0.1612 | 9.78 | <.0001 | 0.05 | 1.2605 | 1.8924 | 4.8378 | 3.5273 | 6.6352 |
| Underlying condition | No | Yes | 2.6691 | 0.2647 | 10.08 | <.0001 | 0.05 | 2.1504 | 3.1878 | 14.4267 | 8.5879 | 24.2352 |
| Income level | 0 to 100% | 101 to 400% of FPL | -0.4711 | 0.2253 | -2.09 | 0.0365 | 0.05 | -0.9126 | -0.02951 | 0.6243 | 0.4015 | 0.9709 |
|  |  | Missing | 0.04728 | 0.1214 | 0.39 | 0.697 | 0.05 | -0.1907 | 0.2852 | 1.0484 | 0.8264 | 1.3301 |
| Household size | 1 person | 2 people | -0.6946 | 0.2822 | -2.46 | 0.0138 | 0.05 | -1.2478 | -0.1414 | 0.4993 | 0.2871 | 0.8681 |
|  |  | 3 people | -0.6863 | 0.2618 | -2.62 | 0.0088 | 0.05 | -1.1994 | -0.1731 | 0.5035 | 0.3014 | 0.841 |
|  |  | 4 people | -0.8981 | 0.2905 | -3.09 | 0.002 | 0.05 | -1.4675 | -0.3286 | 0.4074 | 0.2305 | 0.7199 |
|  |  | 5 or 6 people | -0.943 | 0.2999 | -3.14 | 0.0017 | 0.05 | -1.5307 | -0.3553 | 0.3895 | 0.2164 | 0.701 |
|  |  | 7 or 8 people | 0.1488 | 0.268 | 0.56 | 0.5787 | 0.05 | -0.3764 | 0.674 | 1.1605 | 0.6863 | 1.9621 |
|  |  | Missing | -0.6968 | 0.1391 | -5.01 | <.0001 | 0.05 | -0.9695 | -0.4241 | 0.4982 | 0.3793 | 0.6544 |
| **MarketScan** | | | | | | | | | | | | |
| **Variable** | **Reference group** | **Comparison group** | **Estimate** | **Standard Error** | **z Value** | **Pr > \|z\|** | **Alpha** | **Lower** | **Upper** | **Exponentiated** | **Exponentiated Lower** | **Exponentiated Upper** |
| Age group | 1 | 2 | -1.8484 | 1.0801 | -1.71 | 0.087 | 0.05 | -3.9654 | 0.2686 | 0.1575 | 0.01896 | 1.3081 |
|  |  | 3 | -1.289 | 0.8165 | -1.58 | 0.1144 | 0.05 | -2.8893 | 0.3113 | 0.2755 | 0.05561 | 1.3652 |
|  |  | 4 | 0.3354 | 0.5164 | 0.65 | 0.5161 | 0.05 | -0.6768 | 1.3475 | 1.3984 | 0.5083 | 3.8477 |
|  |  | 5 | -0.3653 | 0.5774 | -0.63 | 0.5269 | 0.05 | -1.4969 | 0.7663 | 0.694 | 0.2238 | 2.1518 |
|  |  | 6 | -0.5936 | 0.6455 | -0.92 | 0.3578 | 0.05 | -1.8588 | 0.6715 | 0.5523 | 0.1559 | 1.9572 |
|  |  | 7 | -2.0254 | 1.0801 | -1.88 | 0.0608 | 0.05 | -4.1424 | 0.09162 | 0.1319 | 0.01588 | 1.096 |
|  |  | 8 | -2.0759 | 1.0801 | -1.92 | 0.0546 | 0.05 | -4.1929 | 0.04114 | 0.1254 | 0.0151 | 1.042 |
|  |  | 9 | -1.3849 | 0.8165 | -1.7 | 0.0899 | 0.05 | -2.9852 | 0.2154 | 0.2503 | 0.05053 | 1.2404 |
|  |  | 10 | -0.8018 | 0.5401 | -1.48 | 0.1376 | 0.05 | -1.8603 | 0.2567 | 0.4485 | 0.1556 | 1.2926 |
|  |  | Missing | -0.7886 | 0.5401 | -1.46 | 0.1442 | 0.05 | -1.8471 | 0.2699 | 0.4545 | 0.1577 | 1.3098 |
| Census division | New England | Middle Atlantic | 0.5076 | 0.7638 | 0.66 | 0.5063 | 0.05 | -0.9894 | 2.0045 | 1.6613 | 0.3718 | 7.4226 |
|  |  | East North Central | -0.5744 | 0.8367 | -0.69 | 0.4923 | 0.05 | -2.2143 | 1.0654 | 0.563 | 0.1092 | 2.9019 |
|  |  | West North Central | -0.4183 | 1 | -0.42 | 0.6758 | 0.05 | -2.3782 | 1.5417 | 0.6582 | 0.09271 | 4.6725 |
|  |  | South Atlantic | -1.4021 | 0.9129 | -1.54 | 0.1245 | 0.05 | -3.1913 | 0.3871 | 0.2461 | 0.04112 | 1.4727 |
|  |  | East South Central | -1.2702 | 1.2247 | -1.04 | 0.2997 | 0.05 | -3.6707 | 1.1302 | 0.2808 | 0.02546 | 3.0964 |
|  |  | West South Central | 0.3816 | 0.7746 | 0.49 | 0.6222 | 0.05 | -1.1366 | 1.8998 | 1.4647 | 0.3209 | 6.6846 |
|  |  | Mountain | -0.1051 | 0.9129 | -0.12 | 0.9083 | 0.05 | -1.8943 | 1.6841 | 0.9002 | 0.1504 | 5.3874 |
|  |  | Pacific | 0.0286 | 0.8165 | 0.04 | 0.9721 | 0.05 | -1.5717 | 1.6289 | 1.029 | 0.2077 | 5.0983 |
|  |  | Missing | 0.05597 | 0.8367 | 0.07 | 0.9467 | 0.05 | -1.5839 | 1.6958 | 1.0576 | 0.2052 | 5.451 |
| Tobacco use | No | Yes | -1.5124 | 0.3865 | -3.91 | <.0001 | 0.05 | -2.27 | -0.7549 | 0.2204 | 0.1033 | 0.4701 |
| Underlying condition | No | Yes | 3.1002 | 0.722 | 4.29 | <.0001 | 0.05 | 1.6851 | 4.5153 | 22.2018 | 5.3929 | 91.4011 |
